# Novel Three-Step Pseudo-Absence Selection Technique for Improved Species Distribution Modelling

**DOI:** 10.1371/journal.pone.0071218

**Published:** 2013-08-13

**Authors:** Senait D. Senay, Susan P. Worner, Takayoshi Ikeda

**Affiliations:** 1 Bio-Protection Research Centre, Lincoln University, Lincoln, New Zealand; 2 University of Otago, Wellington, New Zealand; Bangor University, United Kingdom

## Abstract

Pseudo-absence selection for spatial distribution models (SDMs) is the subject of ongoing investigation. Numerous techniques continue to be developed, and reports of their effectiveness vary. Because the quality of presence and absence data is key for acceptable accuracy of correlative SDM predictions, determining an appropriate method to characterise pseudo-absences for SDM’s is vital. The main methods that are currently used to generate pseudo-absence points are: 1) randomly generated pseudo-absence locations from background data; 2) pseudo-absence locations generated within a delimited geographical distance from recorded presence points; and 3) pseudo-absence locations selected in areas that are environmentally dissimilar from presence points. There is a need for a method that considers both geographical extent and environmental requirements to produce pseudo-absence points that are spatially and ecologically balanced. We use a novel three-step approach that satisfies both spatial and ecological reasons why the target species is likely to find a particular geo-location unsuitable. Step 1 comprises establishing a geographical extent around species presence points from which pseudo-absence points are selected based on analyses of environmental variable importance at different distances. This step gives an ecologically meaningful explanation to the spatial range of background data, as opposed to using an arbitrary radius. Step 2 determines locations that are environmentally dissimilar to the presence points within the distance specified in step one. Step 3 performs K-means clustering to reduce the number of potential pseudo-absences to the desired set by taking the centroids of clusters in the most environmentally dissimilar class identified in step 2. By considering spatial, ecological and environmental aspects, the three-step method identifies appropriate pseudo-absence points for correlative SDMs. We illustrate this method by predicting the New Zealand potential distribution of the Asian tiger mosquito (*Aedes albopictus*) and the Western corn rootworm (*Diabrotica virgifera virgifera*).

## Introduction

Spatial distribution models (SDMs) have been used to model species distribution for conservation, biological control introductions and, particularly, to predict invasive species establishment and spread [1]. Despite some shortcomings, SDMs are very popular. This popularity has largely been driven by greater data availability coupled with increasing sophistication of models as well as computer technology [2], [3], [4]. Correlative SDMs model a species distribution by inferring its environmental niche from known presence locations. Correlative models are popular as the alternatives, mechanistic or process-based models, are not always achievable due to their requirement of extensive knowledge of the environmental and physiological requirements of the species [5], [6]. A major criticism and source of uncertainty in correlative SDM predictions is the lack of true absence information for accurate species distribution predictions [7], [8]. Determining true absences for species distribution prediction is a difficult task. A species could be absent for reasons other than simply because the location is not environmentally suitable [1], [9]. Possible scenarios include: 1) the species has not reached the locality due to natural or human barriers, 2) the species has not been detected despite being present, or 3) it is excluded due to competition. Other potential reasons could also be that the species has become locally extinct despite the environment being favourable or temporarily absent due to migratory behaviour.

Three main approaches are used to compensate for missing absence information. 1) Simple presence-only models, 2) enhanced presence-only models, and 3) presence-absence models. The choice is often influenced by the quality and quantity of presence data and research objectives such as whether a potential or realized species distribution is the target [2].

Simple presence-only models are models that require only presence data to map species distribution or calculate a habitat suitability index. These models constrain environmental requirements for the species to within the extent of the available presence points using various distance or polygon rules to predict the species distribution [10]. Models like BIOCLIM [11] and DOMAIN [12] are good examples of simple presence-only models.

Enhanced presence-only models use presence-only data coupled with background environmental variables and their interactions which are key to understanding the realized niche of the species. These models give a more accurate species distribution prediction than simple presence-only models [2]. Examples of enhanced presence-only models include, maximum entropy (MAXENT) [3], ecological niche factor analysis (ENFA) [9], and the presence and background learning algorithm (PBL) [13]. All presence-only models are sensitive to biases in presence data as all information for the species distribution is primarily dependent on the presence points. Background sampling in presence-only models is often mistaken for selecting pseudo-absence points. However, background data sampling (instead of using the whole background) in such models is usually done to shorten computation time when using large or very high resolution datasets [3], [14].

Presence-absence models use both presence and absence information to predict habitat suitability and/or species distribution. In cases where real absences are not available, various techniques are used to generate pseudo-absence points. There are a number of models used for presence-absence modelling. Some are regression based models like generalized linear models (GLM) and generalized additive models (GAM) that have been frequently used. While other novel machine learning and classification models like artificial neural networks (ANN), support vector machines (SVM) and naïve Bayes (NB) have only recently been used for ecological modelling [15], [16]. These models can be roughly classified based on their algorithms as regression, classification and machine learning. This is not a strict category as some models mix various types of algorithms. One characteristic presence-absence models have in common is that a set of true or pseudo-absence locations are needed to model habitat suitability or species distributions.

The disagreement among studies that have evaluated these three types of models [3], [15], [17], [18], [19], [20], [21] shows that each type has merits depending on the modelling context, such as: availability of presence data, characteristics of the predictor data and the modelling expertise available. Presence-only models work best when there is a reasonable sample of presence information for the target species, preferably with minimal bias [3], [9]. If the available presence data is incomplete or uncertain, presence-absence models are thought to produce more robust results. That is because absence and/or pseudo-absence points can minimize over-prediction and extrapolation into unknown areas [15], [22]. It is always better, statistically, to develop a model that predicts based on negatives (in our case absences or zeros) and positives (presences or ones) than only using positives, provided that the negative data are reliable [23]. Availability of true absence points is very limited in reality, thus to obtain the advantage of presence-absence models reliable pseudo-absences are required. A number of studies have proposed different, often contradicting pseudo-absence selection methods [4], [7], [10], [24], [25], [26]. Even with contrasting recommendations about pseudo-absence selection methods, these studies agree that the quality of pseudo-absence data directly affects the accuracy of model predictions.

### Types of Pseudo-absence Selection Methods

#### Simple random pseudo-absence selection

This method involves taking pseudo-absence points from the background data at random usually excluding known presence points. No prior information about the presence and background data is incorporated to the selection procedure [7], [27]. A variation of this method is when available true absence records are included along with the selected random pseudo-absence points [28].

#### Pseudo absence points with limited geographical extent

This method involves selection of pseudo absence points within (or outside) a certain geographic distance from presence points. Some studies use trial and error where pseudo-absence locations are selected from an area encompassed by varying radii around known presence points. The ideal distance (radius) is chosen based on model performance results [4], [10], [19], [24]. There are also cases where the radius is chosen arbitrarily or based on expert knowledge about the species [17].

#### Pseudo-absence points based on environmental variables

Models that use this method are often referred to as a two-step-pseudo absence selection method. The method involves prior profiling of environmental data into classes [20], [25], [29] using niche analysis models such as ENFA, MDE [30], BIOCLIM [31], statistical methods like the Poisson point process method [26], or simply removal of the known environmentally suitable locations from background data before selecting pseudo-absences. Once the least suitable areas are identified by such profiling, pseudo-absence points are selected at random. Many studies report increased accuracy using this approach. Moreover, judging from recent studies [25], [26], [32] it seems this method has become a standard.

To recapitulate, current pseudo-absence selection methods either optimize for better environmental or spatial discrimination. There is no existing method that provides a balance between these two dimensions. Good discrimination between presence and pseudo-absence points in environmental space alone gives models clear information about the domains in which the species could or could not occur. However, if there is no spatial constraint a model is likely to pick up global or larger scale differences rather than local variations that result in “there-are-no-polar-bears-in-the-Sahara” predictions [4]. VanDerWal *et al.* [24] reported that geographical/spatial extents of background data affected the accuracy of model predictions for 12 species. Furthermore, variable importance varied depending on the size and extent of background data [24]. This result raises two important questions. Does bounding background data at a certain distance from the presence points before pseudo-absence selection affect prediction accuracy? If so, what distance is appropriate for the species and predictor variables involved? According to Lobo *et al.* [4] decisions about giving either spatial or environmental space more weight while selecting pseudo-absence points depends on whether the objective of the study is to model the realized or potential distribution of the target species. This study progresses the ideas proposed by Lobo *et al.* [4] and provides a tested protocol that incorporates the use of geographically and environmentally balanced pseudo-absence points for improved habitat suitability analysis. The full geographic and environmental range of species in the early stages of invasion is usually unknown, especially of those transported globally through trade or tourism. This novel pseudo-absence selection method will be especially useful for modelling species distributions of invasive species at either a global or regional level. In this study, comparisons are made between model predictions based on the three-step method and predictions that used the three commonly used pseudo-absence selection techniques. Presence data for two species with varying relative occurrence area were used for this research. A separate habitat suitability projection is also made for New Zealand, an area which was masked out from the global data to investigate the effect of pseudo-absence methods on model habitat suitability projections using independent data. New Zealand was chosen because the species modelled are not currently established in that country. The first set of presence points is for the species *Aedes albopictus,* (Skuse) (Diptera, Culicidae) commonly known as Asian tiger mosquito. Native to south-east Asia, *A. albopictus* has invaded the Americas, Indo-Pacific regions, Australia, Europe and Africa [33]. *A. albopictus* has invaded a wide range of environments and is a vector of at least 22 arboviruses known to cause diseases in humans and animals [34], [35]. The second set of presence points is for the subspecies *Diabrotica virgifera virgifera* (LeConte) (Coleoptera: Chrysomelidae, Galerucinae) commonly known as the western corn rootworm (WCR). *D. v. virgifera* is a known pest of maize plantations mainly in North America and Mexico. According to Coats *et al.* [36], the pest is likely to have been introduced to the North American continent about 1,000 years ago from its tropical native origin in Central America. North America is now considered a native range and source of the recent *D. v. virgifera* introductions to Europe [37], [38], [39], [40]. *D. v. virgifera* is currently a high risk invasive species, partly because of its recent rapid range and host expansion throughout Europe, and due to its relatively quick adaptation to overcome pest control practices [41], [42].

Objectives of this study are to determine: 1) whether pseudo-absences optimised for both the spatial and environmental range of the species increase model performance and accuracy; and 2) whether different pseudo-absence selection methods affect models differentially.

## Methods

### Biotic Data

**Figure 1:**
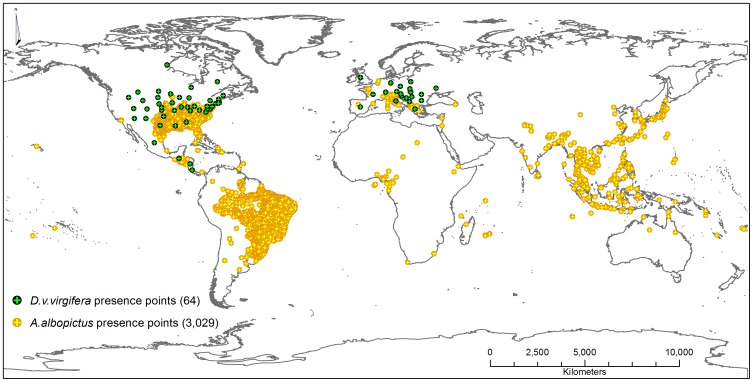
Map of global presence data for *A. albopictus* and *D. v. virgifera.*

The target species *A. albopictus* and sub-species *D. v. virgifera* were chosen for their different relative occurrence area (ROA) in both geographic (Figure 1) and environmental space. Because *A. albopictus* is a critical health hazard, extensive research has been undertaken in areas of insect control, such that there were 3,029 presence points available for this study acquired from literature, personal communication with experts and CABI and GBIF databases (Ikeda *et al.* unpublished data). Out of the 3,029 presence points, 2,928 were spatially unique with respect to the resolution of the environmental data used in this study. For *D. v. virgifera,* there were 64 presence points available for this study (data courtesy of GBIF and PRATIQUE). All *D. v. virgifera* points were used for modelling as they were all spatially unique with respect to the data resolution of the environmental layers.

### Environmental Data

Data from the BIOCLIM dataset [43] which is derived from a 50-year-average (1950–2000) daily temperature and precipitation dataset (WORLDCLIM) [44] prepared in 10 arc minute (0.17°) resolution [43] was used to source the 19 bioclimatic variables shown in Table 1. A geographical variable, elevation, was also obtained through the BIOCLIM data portal. Hijmans *et al.* [44] reported that the bulk of the elevation dataset was sourced from NASA’s SRTM [45] global Digital Elevation Model with additional data from GTOPO30 [46] global elevation data to cover the above 60°N areas for which there was no SRTM data. Elevation is known to moderate local climate and it could act as a natural barrier between suitable areas. Elevation was added to account for local topographical variations in habitats.

**Table 1:** List of variables selected using four pseudo-absence selection methods for the two target species.

No.	Variables	aaSM1	aaSM2	aaSM3	aaSM4	dvvSM1	dvvSM2	dvvSM3	dvvSM4
V1	Annual Mean Temperature	✓	✓	✓	✓		✓	✓	
V2	Mean Diurnal Range (Mean of monthly (max temp - min temp))							✓	
V3	Isothermality (P2/P7) (* 100)	✓	✓	✓		✓	✓	✓	
V4	Temperature Seasonality (standard deviation *100)	✓	✓	✓					
V5	Max Temperature of Warmest Month	✓		✓	✓			✓	
V6	Min Temperature of Coldest Month	✓		✓		✓		✓	
V7	Temperature Annual Range (P5–P6)	✓	✓						
V8	Mean Temperature of Wettest Quarter			✓					
V9	Mean Temperature of Driest Quarter	✓		✓					
V10	Mean Temperature of Warmest Quarter	✓	✓	✓	✓			✓	
V11	Mean Temperature of Coldest Quarter	✓	✓	✓		✓		✓	
V12	Annual Precipitation	✓	✓	✓	✓			✓	
V13	Precipitation of Wettest Month	✓	✓	✓	✓			✓	
V14	Precipitation of Driest Month	✓						✓	✓
V15	Precipitation Seasonality (Coefficient of Variation)	✓	✓		✓			✓	
V16	Precipitation of Wettest Quarter	✓	✓	✓				✓	✓
V17	Precipitation of Driest Quarter	✓	✓	✓	✓		✓	✓	
V18	Precipitation of Warmest Quarter						✓		
V19	Precipitation of Coldest Quarter	✓							
V20	Altitude	✓		✓	✓				
Total		17	11	14	8	3	4	13	2

* aa = *Aedes albopictus*, dvv = *Diabrotica v. virgifera*, SM1 = random pseudo-absence selection method, SM2 = spatially constrained random pseudo-absence selection method, SM3 = 2-step environmental profiling pseudo-absence selection method, SM4 = 3-step environmental profiling with spatial constraint pseudo-absence selection method.

Two of the pseudo-absence selection methods in this study use plane circular buffers on background data to limit the pseudo-absence selection within a certain distance from presences. Such planar buffers cannot be overlaid on data in the geographic coordinate system without causing poleward distortion. To avoid this bias, the global (0.17°) and New Zealand extent (30″) data were converted into world Mercator WGS 1984 coordinate system and UTM-WGS1984-Zone-59S coordinate system respectively. Both datasets are then resampled into a 15.2 km×15.2 km and 0.8 km×0.8 km equal area grids using bilinear interpolation. Optimum cell sizes were determined as follows.

For the global data, the vertical range of the BIOCLIM data (82°N∼56°S) was used to define latitudinal ranges of 40°N∼40°S, between 40°N∼60°N & 40°S∼56°S, and greater than 60°N. The optimum cell size was identified by weighting the average of the mean cell width in each pre-determined latitudinal range by the number of pixels in the latitudinal range. Weighting along latitudinal zones was not necessary for the New Zealand data as the change in horizontal cell size along latitude was small (∼0.02 km). The cell size for New Zealand was calculated by taking the square root of the product of the average cell width (0.71 km) and average cell height (0.93 km) in the dataset.

All of the 20 variables were combined in one raster dataset with multiple attributes and converted into a vector point dataset, which was then exported into an ASCII matrix. Each point in the matrix represented an area of 231.3 km^2^ within the global data set and an area of 0.64 km^2^ within the New Zealand dataset. The total area of analysis covers all global landmass except Antarctica with an area of 135,202,962 km^2^. The New Zealand data covered 268,042 km^2^.

A non-New Zealand global dataset was used as a background for pseudo-absence selection. This is done to provide all models with a standardized independent dataset (New Zealand) which is used for habitat suitability projections. An environmental similarity test was undertaken by mapping the New Zealand extent in the environmental feature space of PCA transformed BIOCLIM data. There were no New Zealand data points outside the environmental bounds of data for the rest of the world, ensuring models did not extrapolate. The full extent global data was used for global habitat suitability predictions, and the high resolution data was used for habitat suitability projections in New Zealand.

### Simple Random Pseudo-absence Selection (SM1)

Pseudo-absence points are selected randomly from across the whole study area. Known presence points were removed prior to random selection making the size of the background data 134,515,972 km^2^ for *A. albopictus* and 135,184,400 km^2^ for *D. v. virgifera*. The ratio of presence data to pseudo-absence data is debated [4], [10], [20], [28]. An unbalanced design where there are more pseudo-absence points than presence points has been found to affect performance of some models positively, and others negatively [10]. That introduces bias in research designs involving multiple models such as this study. Therefore, an equal number of pseudo-absence points as presences points were used for the random selection method and all subsequent pseudo-absence selection methods used in this study. Random 2,928 and 64 points were selected for *A. albopictus* and *D.v.virgifera* respectively from the background data.

### Spatially Constrained Pseudo-absence Points Selection (SM2)

This method uses a spatial constraint on background data before selecting pseudo-absence points. The background data is extracted within a defined distance from presence points. Previous applications of this method have often used an arbitrarily chosen distance [24]. Pseudo-absence points were then chosen at random from the geographically limited background data. For consistency, in our study the same distances determined within the 3-step method were used. These distances were 350 km for *A. albopictus* and 3,000 km for *D. v. virgifera*. Pseudo-absence points were selected at random from the spatially constrained background dataset. The background data set for this scenario covered 29,219,485 km^2^ for *A. albopictus* and 64,791,235 km^2^ for *D. v. virgifera.*

### Environmental Pseudo-absences Point Selection (SM3)

An environmental profiling, similar to other two-step pseudo-absence generation methods [7], [25] was performed on the background data except that a one class support vector machine (OCSVM) [47] classifier was used. OCSVM is chosen because it can handle high dimensional data and complex non-linear relationships between predictors. The OCSVM model was trained with environmental variable data at presence points. An ensemble of 100 best performing OCSVM models was used to determine robust environmentally profiled background classes (Ikeda *et al.* unpublished data). Using an ensemble approach rather than the single best performing model reduced the probability of choosing an over-fitted model. The OCSVM profiling produced background data with values between zero and one, which represent the probability of being similar to the presence data. All background data points with a probability of 0 (zero-similarity with presences) were extracted as potential pseudo-absence points. Random 2,928 and 64 pseudo-absences were selected from this zero-similarity background data that covered 102,831,933 km^2^ and 87,744,064 km^2^ for *A. albopictus* and *D. v. virgifera* respectively.

### Three Step Pseudo-absence Selection Method (SM4)

The novel three-step method developed here provides a balance between using the spatial and environmental space for selection of appropriate pseudo-absence points. The first step is to determine geographic space for the species by establishing the appropriate distance by which background data is bound to presence data. In the second step, an OCSVM model is used to classify the background data constrained in step 1 into various environmental classes. In the third step K-means clustering is used to select a representative sample from all the environmentally dissimilar points identified in step 2 as pseudo-absence points.

#### Step 1: Specifying geographical extent

An independent method based on variable importance analysis was designed to identify an appropriate distance by which background data is bounded to presence points. First, multiple datasets were produced by bounding background data at different radii from presence points. We chose 50 km, 100 km, 150 km, 200 km, 250 km, 300 km, 350 km, 400 km, and 500 km intervals to test for change in variable importance (Figure S1). In cases where no change was observed within the listed intervals the distance was increased by 100 km until change was observed. Variable importance was analysed by performing principal component analysis (PCA) on these different background datasets. Variables that contribute the most (up to 70%) to the first component were identified. The contribution of these variables versus distance was then plotted and analysed for any decline in contribution to the first principal component. The distance at which the contribution of the most important variables declined or stopped increasing was chosen as the optimal limit to bound background data. We suggest that including background data outside the optimum distance could obscure important information for feature selection. Tuv *et al.* [48] and references therein show that unnecessarily large and redundant background data introduces noise and decreases predictive power of models. The contribution of the most important variables to the first principal component declined at 350 km for *A. albopictus*, and at 3,000 km for *D. v. virgifera*, these distances were taken as the optimum boundary of background data. The area of the background data extracted from within the optimum distance of presence points was 29,219,485 km^2^ for *A. albopictus* and 64,791,235 km^2^ for *D. v. virgifera*.

#### Step 2: Environmental profiling of background data

Environmental profiling was performed on the spatially limited background data identified at step 1 using an OCSVM [47] classifier. All locations with a probability of 0 (zero similarity with presence points) were extracted as a potential background for pseudo-absence selection. This procedure further reduced the background data at step 1 to 9,925,310 km^2^ and 12,878,516 km^2^ for *A. albopictus* and *D. v. virgifera* respectively.

#### Step 3: K-means clustering

K-means clustering was used to group the zero-similarity locations defined at step 2 into k clusters according to their environmental value. The parameter k that determines the number of clusters for K-means clustering was set to the number of presences available (K = 2,928 for *A. albopictus* and K = 64 for *D. v. virgifera*). The centroids, from each cluster in the environmental feature space, were selected as they best represented their respective cluster. The projection of the centroids in the geographic space provided the pseudo-absence points needed to proceed with the presence–absence modelling.

### Model Evaluation and Output Analysis

The four methods of pseudo-absence selection were compared based on the performance of seven presence-absence models. The seven models were 1) logistic regression (LOG) [49], 2) classification and regression trees (CART) [50] 3) conditional trees (CTREE) [51]. 4) K-nearest neighbours (KNN) [52]; 5) naïve Bayes (NB) [53], 6) support vector machines (SVM) [54] and 7) artificial neural networks (NNET) [55].

Variable selection was carried out using random forests. Random forest (RF) is a classification algorithm that uses an ensemble of classification trees. Random forest is chosen because it does not overfit and also because it is reported to have a good predictive performance even when noisy variables are included [56]. Variable selection was performed independently for each training dataset, as the domain and range of the four types of pseudo-absences vary in the geo-environmental space. Table 1 shows the list of variables selected for the different scenarios. For validation, 20% of both the presence and pseudo-absence datasets were partitioned and set aside for cross-validation while 80% was used to train the models. Performance of each model was measured after 10-fold cross-validation with 20 repetitions. The models were compared based on performance scoring methods (Table 2).

**Table 2:** Model performance indices.

Index		Abbreviations	Remark
***Accuracy = ***	(TP+TN)/(TP+TN+FP+FN)	TP = True positive; TN = True negative	
		FP = False positive; FN = False negative	
***Kappa = ***	((OA-EA))/(((TP+FP+TN+FN)-EA))	OA = observed agreement (Accuracy)	Values > = 0.81 = Excellent;
	Where EA =	EA = Expected agreement	0.61–0.80 = Good; 0.41–0.60 = Medium;
	(TP+FN)(TP+FP)/(TP+FP+TN+FN) −		0.21–0.40 = Not good; 0.00–0.20 = Bad;
	(FP+TN)(TN+TN+FN)/(TP+FP+TN+FN)		Values <0.00 = Very bad
***Sensitivity = ***	TP/(TP+FN)		1 - omission error (recall)
***Specificity = ***	TN/(TN+FP)		1 - commission error
***AUC = ***	A plot of TPR vs. FPR	AUC = Area under the ROC* curve	Calculated on the test dataset
	Where FPR = FP/(FP+TN)	TPR = True positive rate (sensitivity)	Values >0.7 are considered good
		FPR = False positive rate	

* ROC: Receiver operating characteristic.

The threshold p>0.5 was used to convert model predictions into binary presence-absence maps to obtain predicted presences. There is evidence that shows predefined thresholds such as used in this study may lead to a cut-off that does not approximate the true threshold at which the species is likely to be present [57]. The optimum threshold based on prevalence is considered to decrease towards zero for rare species and increases towards one for generalist species [57]. Both species used in this research are not rare; thus the bias introduced from erroneous threshold should be similar. The threshold of 0.5 was used as we were interested solely in variation arising from pseudo-absence selection methods. Percentages of predicted presences out of the total study area were compared for differences in habitat suitability predictions among models using the different pseudo-absence methods. Model consensus was analysed for the New Zealand extent, by identifying how many models using the same pseudo-absence method predicted similarly over their respective predicted presence maps.

A habitat suitability prediction was produced using the best model for each pseudo-absence selection scenario at the global extent and for New Zealand. Model kappa values were used to select the best model for each pseudo-absence method scenario. Kappa is chosen because it is a robust performance index which corrects for prediction success by chance [58]. Habitat suitability maps are re-projected back onto a geographic co-ordinate system for visualization.

All analyses were carried out using the free software R [59] version 2.8.1 and 2.15.1 with packages agricolae [60], class, nnet, MASS [61], Coin [62], e1071 [63], kernlab [64], klaR [65], multcomp [66], randomForest [67], SP [68], VarSelRF [69]. The R software based multi-model framework programmed by Ikeda *et al.* (unpublished data) was used to run the models in a standardized manner. Data pre-processing and mapping were done using MATLAB version R2011a [70] and ArcGIS version 10.1 [71].

## Results

### Pseudo-absences

**Figure 2:**
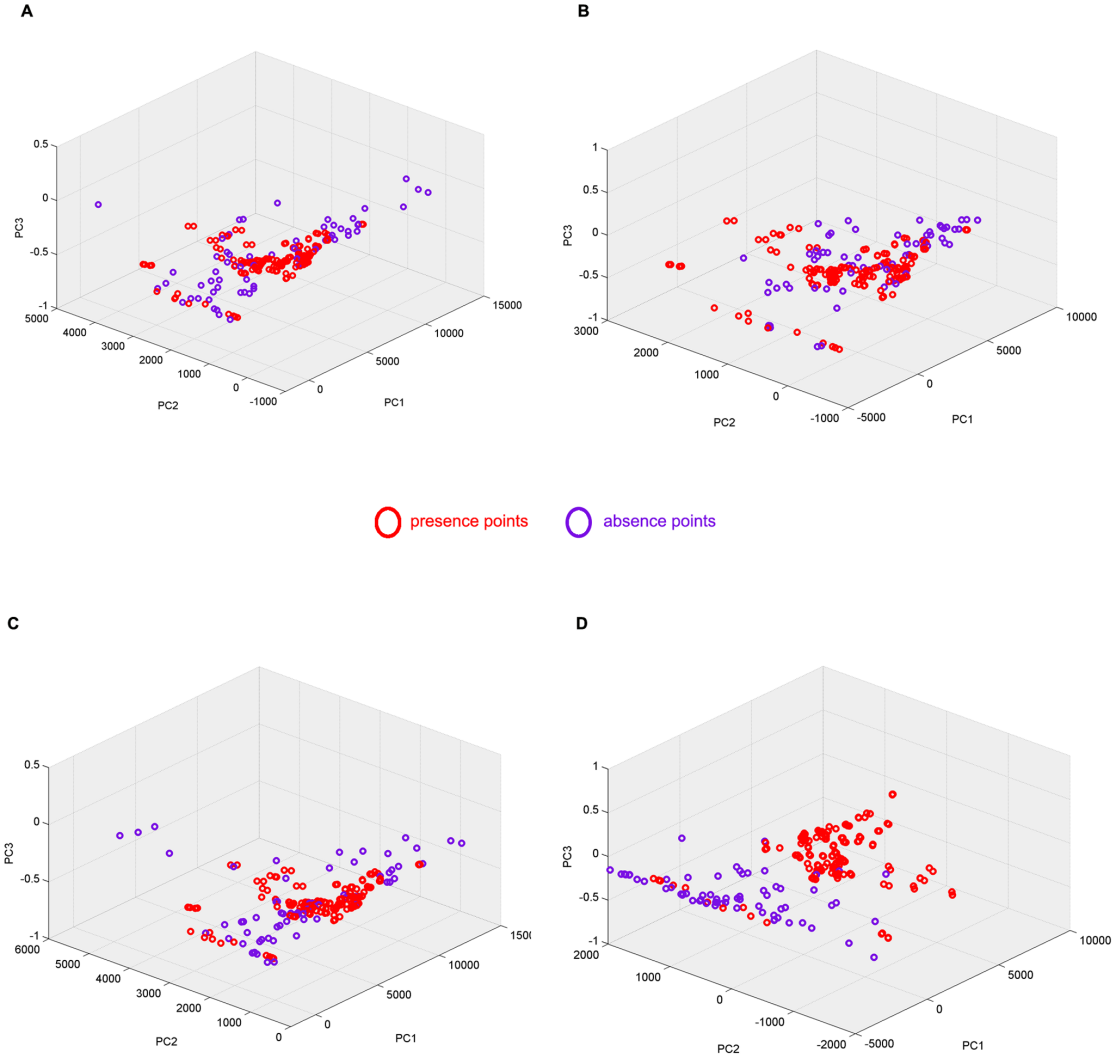
Pseudo-absence points from the four pseudo-absence selection methods. Pseudo-absence points plotted with presence points on the first three principal components of the training dataset (Species: *D. v. virgifera),* (A) SM1, (B) SM2, (C) SM3, and (D) SM4.

The environmental range and domain of pseudo-absences from the 4 pseudo-absence selection methods were different for both species (Figure 2). Only *D. v. virgifera* training data points were plotted as the large number of presence points for *A. albopictus* made the plot unintelligible. SM1 pseudo-absences were closely clustered around presence points with only a few points discriminated from presence points in the environmental feature space (Figure 2a). SM2 pseudo-absences were also closely clustered around presence points (Figure 2b). SM3 (Figure 2c) and SM4 pseudo-absences points (Figure 2d) were clearly discriminated from presence points. However, the SM4 pseudo-absences were environmentally further from presence points than the other 3 methods. This is illustrated by their magnitudes on the principal component axes (Figure 2).

### Variable Selection

There was considerable variation in the subset of variables chosen for each training dataset from the total predictor list of 20. The 3-step selection method (SM4) gave fewer variables for both *A. albopictus* and *D. v. virgifera* (Table 1).

### Model Performance

**Figure 3:**
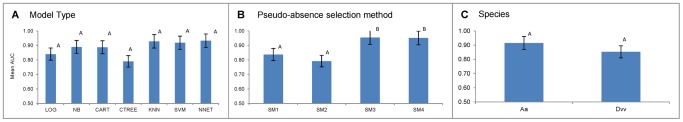
Variation of mean AUC values due to model type, pseudo-absence selection method and number and structure of presence data. Error bars indicate standard errors over replicates. Bars with same letters within a graph are not significantly different (Tukey’s HSD test p>0.05). (A) model type, (B) pseudo-absence selection method, and, (C) species dataset.

Out of the 56 models from the various data-method-model combinations (7 model types×4 selection methods×2 species), 55 of the models had mean AUC value better than 0.5 meaning all models predicted better than chance except for one model (CTREE,SM1,*Dvv*), which registered a poor performance (AUC = 0.1765). Two-way within subjects analysis of variance was used to calculate the variance attributed to each factor in the experiment. The pseudo-absence selection method had a highly significant (ANOVA, p = 0.0017) effect on model mean AUC values, but the interaction between model type and selection method was insignificant. Mean AUC differences due to model type were not significant according to Tukey’s HSD test (p<0.05). There was a statistically significant difference in mean AUC of models using SM1 and SM2 pseudo-absences compared with models using SM3 and SM4 pseudo-absences (p<0.05) (Figure 3). The average mean AUC of models using SM1, SM2, SM3 and SM4 pseudo-absence points was 0.84 (±0.21 SD), 0.79 (±0.07 SD), 0.95 (±0.05 SD), and 0.95 (±0.03 SD) respectively.

We used the proportion of the sum of correctly predicted pseudo-absences and correctly predicted presences out of the total test data to calculate model accuracy. The ANOVA results for the mean accuracy values for the same models under different pseudo-absence selection methods showed that pseudo-absence selection method has a significant effect on model accuracy (p = 0.00002). Tukey’s HSD test on model accuracy measurements also gave a similar result to comparison of mean AUC values; models using pseudo-absence selection methods SM3 and SM4 have significantly better accuracy than models that used SM1 and SM2 pseudo-absences (p<0.05).

### Prediction-reality Agreement

**Figure 4:**
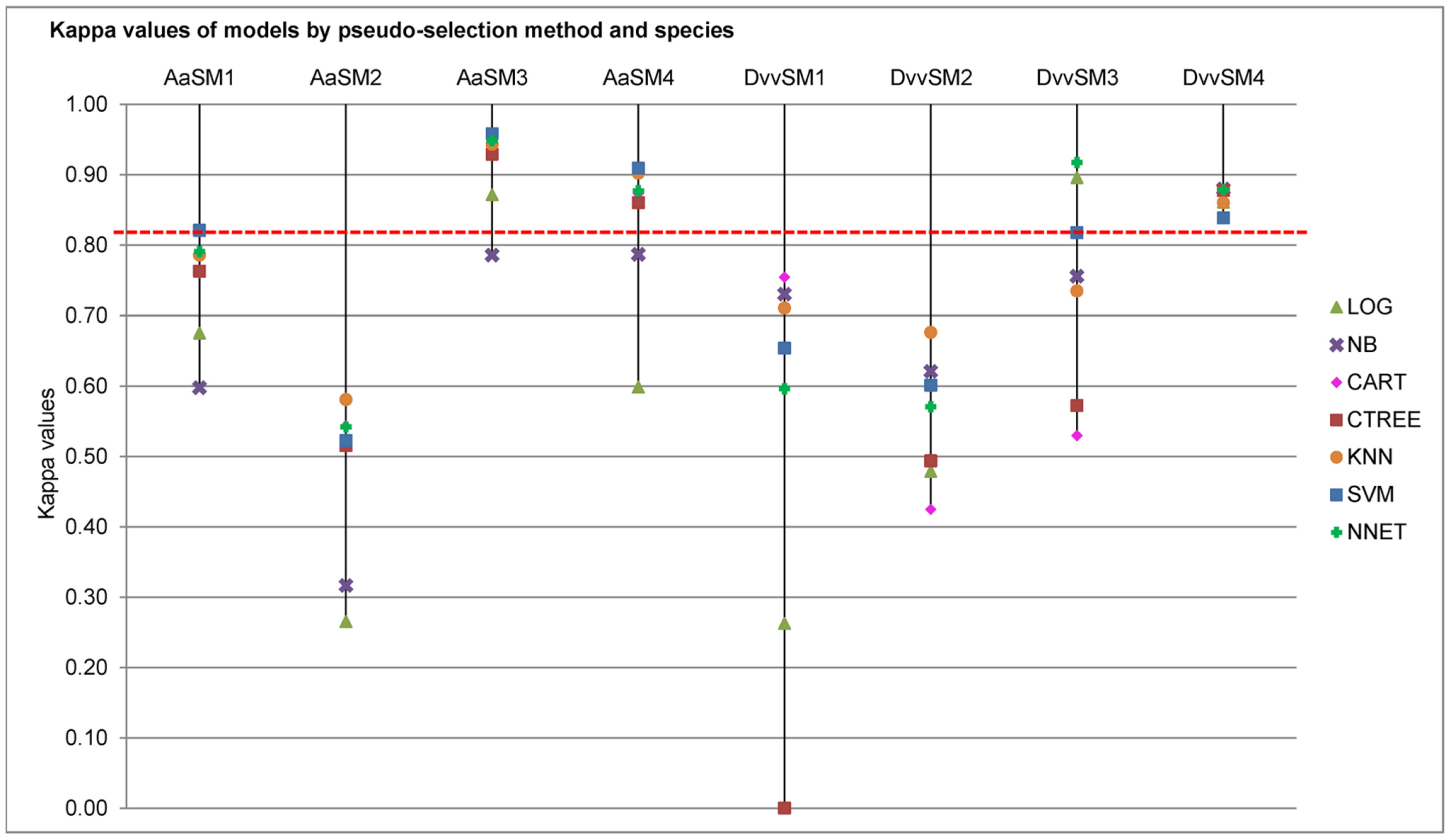
Kappa values of models for the four pseudo-absence selection methods and two species datasets. *Aa = A. albopictus*, Dvv = *D. v. virgifera*, values above the red broken line are in the excellent band of the kappa index.

The Kappa index was used to compare results between the different models according to pseudo-absence selection method. SM1 resulted in 13 out of the 14 models that were between ‘good – bad’ bands with the exception of one model (SVM, SM1, *A. a*) in the ‘excellent’ band (Figure 4). The range of scores for the SM1 method was between 0.59 - 0.82 for *A. albopictus* and 0.00 - 0.75 for *D. v. virgifera*. For method SM2, none of the 14 tested models-species combinations were in the ‘excellent’ band with Kappa values between 0.43 - 0.58 over the two species. For method SM3, model Kappa scores were in the ‘excellent’ band for 9 out of 14 models and in the ‘medium to good’ bands for the remaining 5 models over the two species. For method SM4 model Kappa scores were in the ‘excellent’ band for 12 out of 14 models and in the ‘good’ and ‘medium’ bands for the remaining two models over the two species (Figure 4).

### Specificity and Sensitivity

**Figure 5:**
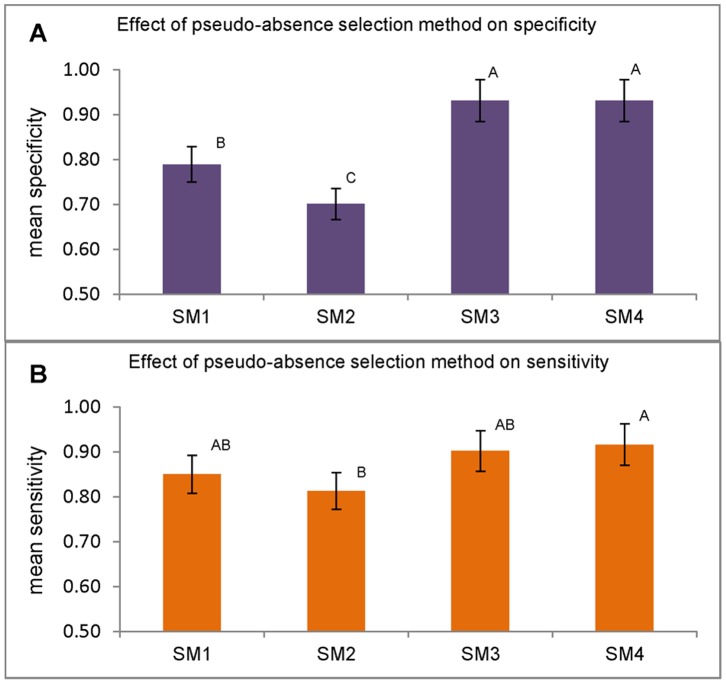
The effect of pseudo-absence selection method on mean specificity and sensitivity values. Error bars indicate standard errors. Bars with same letters are not significantly different (Tukey’s HSD test p>0.05), (A) specificity (B) sensitivity.

Analysis of variance of the specificity results of the seven models showed that there is a highly significant difference between specificity scores of models using different pseudo-absence selection methods (p<0.0001) over the two species, and the model type also had a significant contribution towards the variation in the specificity results (p = 0.011). The lowest mean specificity values were obtained from models using pseudo-absence selection method SM2, models using SM1 pseudo-absence points also had low specificity scores but were significantly better than SM2 models (Figure 5). Models that used SM3 and SM4 pseudo-absence points gave significantly better specificity than SM1 and SM2. There was a similar trend for sensitivity where the pseudo-absence selection method had a significant effect on model sensitivity (p = 0.025). All models with SM3 and SM4 pseudo-absences scored high sensitivity values (>0.85) for both species dataset (SM3, mean = 0.90, SD = ±0.10; SM4, mean = 0.91, SD = ±0.02). While models with SM1 and SM2 pseudo-absences had low sensitivity scores (SM1, mean 0.85, SD = ±0.14; SM2, mean 0.81, SD = ±0.05). There was a considerable between-species variation with respect to sensitivity scores of models using SM1 and SM2.

### Predicted Presence, Model Consensus and Habitat Suitability

There were variations with respect to the number and location of predicted presences by models that used the four different pseudo-absence selection methods. For the global analysis, models using the SM4 method resulted in the highest percentage of predicted presences (mean = 39.29%, SD = ±17.65), with SM2 and SM3 ranking second (mean = 31.70%, SD = ±18.24) and third (mean = 24.82%, SD = ±10.19) respectively while SM1 (mean = 22.77%, SD = ±11.15) gave the smallest percentage of predicted presences. For the New Zealand data, methods SM2 (mean = 52.42%, SD = ±30.27), SM3 (mean = 50.30%, SD = ±37.11), and SM4 (mean = 51.81%, SD = ±29.68), gave very similar predicted presence percentages. The percentage of predicted presences from models using SM1 pseudo-absences was significantly lower (mean = 9.90%, SD = ±17.78) than models using all the other three methods (p = 0.01, p = 0.02, p = 0.01, Tukey’s HSD test in comparison with SM2, SM3 and SM4 respectively).

**Figure 6:**
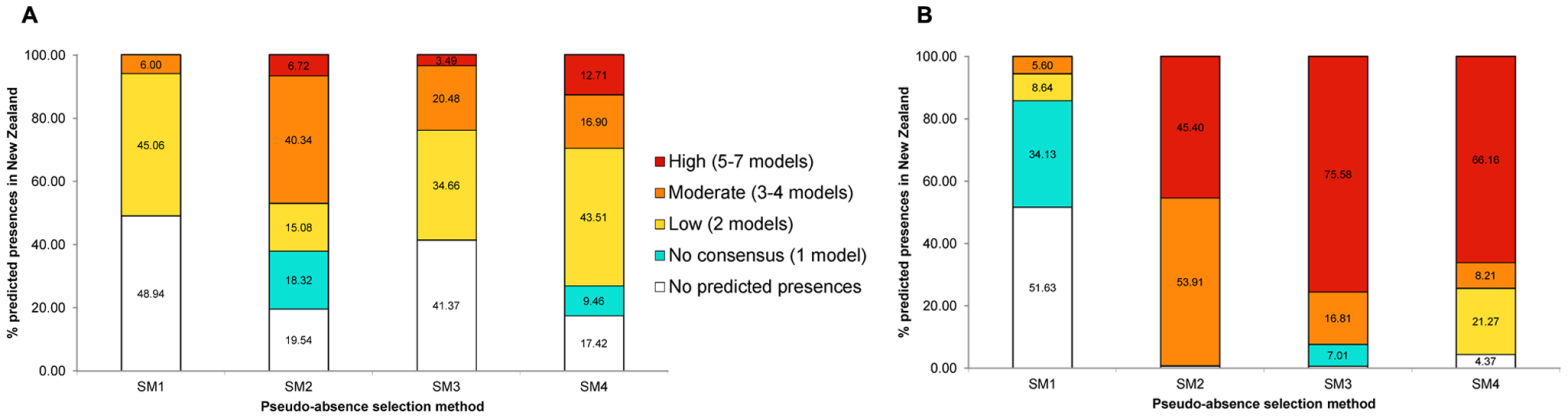
Percentages of predicted presences and respective model consensus on predictions in New Zealand. (A), Asian tiger mosquito (*A. albopictus).* (B), Western corn rootworm (*D. v. virgifera*).

The predicted presences for both *A. albopictus* and *D. v. virgifera* in New Zealand were analysed to investigate the level of model consensus in the predictions. Model consensus was categorized as follows; prediction by 1 model = no consensus, prediction by 2 models = low consensus, prediction by 3–4 models = moderate consensus, and prediction by 5–7 models = high consensus. Predicted presence percentages and model consensus levels are given in figure 6.

**Figure 7:**
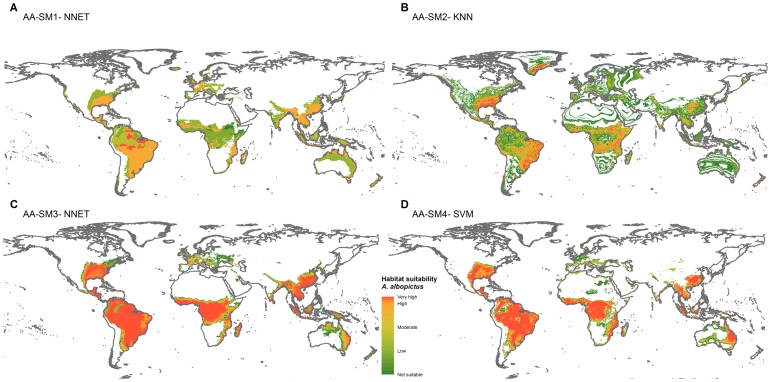
Global habitat suitability prediction for Asian tiger mosquito (*A. albopictus*). (A), SM1 pseudo-absences with model NNET *(B)* SM2 pseudo-absences with model KNN (C), SM3 pseudo-absences with model NNET (D) SM4 pseudo-absences with model SVM. Note: *A. albopictus* occurrence data is too dense to overlay on prediction map, refer to Figure 1. Legend key: not suitable = p<0.4, low = 0.4<p<0.5, moderate = 0.5<p<0.7, high = 0.7<p<0.9, very high = p>0.9.

**Figure 8:**
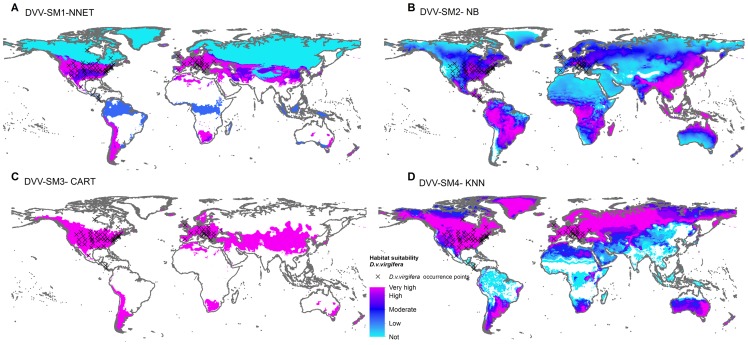
Global habitat suitability prediction for Western corn rootworm (*D. v. virgifera*). *(A)*, SM1 pseudo-absences with model NNET *(B)* SM2 pseudo-absences with model NB (C), SM3 pseudo-absences with model CART (D) SM4 pseudo-absences with model KNN. Legend key: not suitable = p<0.4, low = 0.4<p<0.5, moderate = 0.5<p<0.7, high = 0.7<p<0.9, very high = p>0.9.

Habitat suitability maps were produced using the best models, according to Kappa score, for each scenario. For the *A. albopictus* dataset, the best performing models based on SM1, SM2, SM3 and SM4 pseudo-absence methods were NNET, KNN, NNET and SVM respectively. For the *D. v. virgifera* dataset, the best performing models based on SM1, SM2, SM3 and SM4 pseudo-absences methods were NNET, NB, CART, and KNN respectively (Figures 7 & 8).

**Figure 9:**
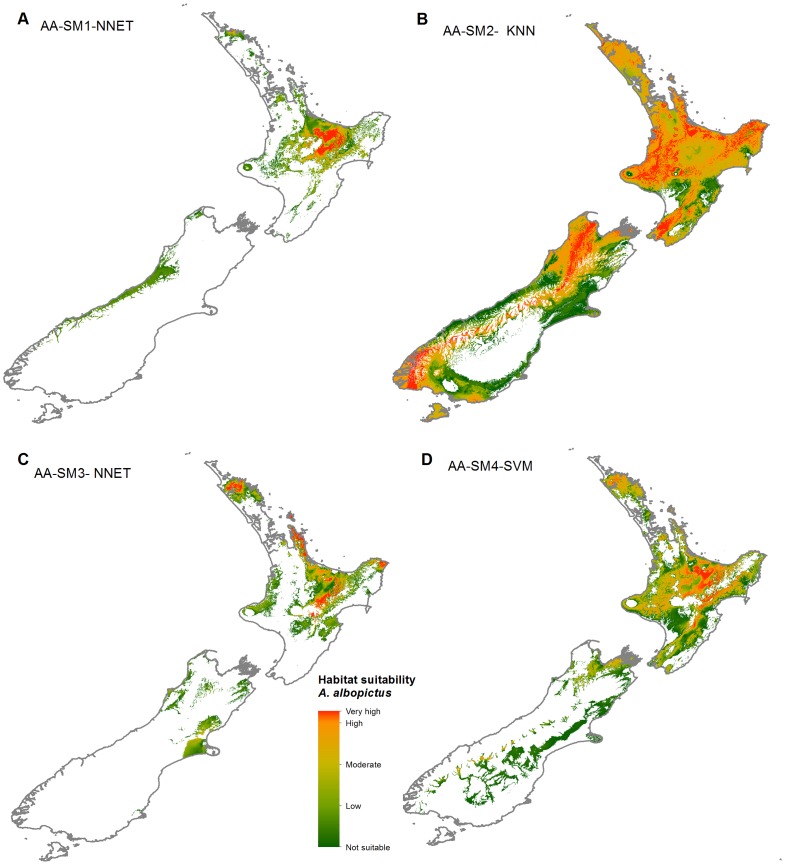
Habitat suitability prediction for Asian tiger mosquito (*A. albopictus*) in New Zealand. *(A)*, SM1 pseudo-absences with model NNET *(B)* SM2 pseudo-absences with model KNN (C), SM3 pseudo-absences with model NNET (D) SM4 pseudo-absences with model SVM. Legend key: not suitable = p<0.4, low = 0.4<p<0.5, moderate = 0.5<p<0.7, high = 0.7<p<0.9, very high = p>0.9.

**Figure 10:**
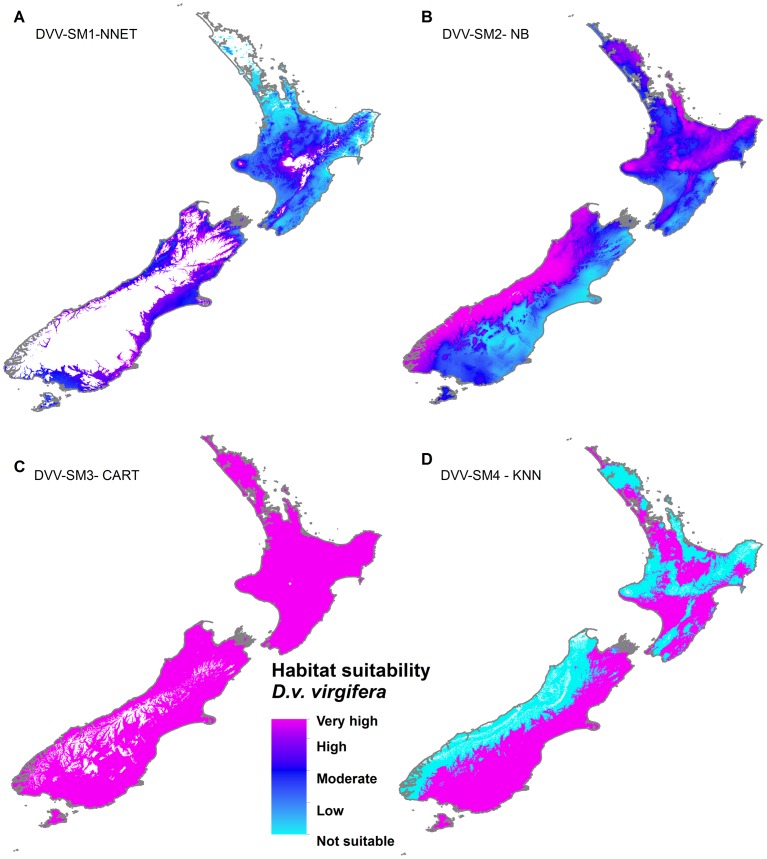
Habitat suitability prediction for Western corn rootworm (*D. v. virgifera*) in New Zealand. *(A)*, SM1 pseudo-absences with model NNET *(B)* SM2 pseudo-absences with model NB (C), SM3 pseudo-absences with model CART (D) SM4 pseudo-absences with model KNN. Legend key: not suitable = p<0.4, low = 0.4<p<0.5, moderate = 0.5<p<0.7, high = 0.7<p<0.9, very high = p>0.9.

Habitat suitability map comparisons in the projections range show that SM1 based maps were dissimilar from SM2, SM3 and SM4 suitability maps (Figure 9, 10). The SM1 suitability predictions both for *A. albopictus* and *D. v. virgifera* in New Zealand were limited to very small areas of low to moderate suitability. The habitat suitability projected using SM2 pseudo-absences identified 72,557 km^2^ of highly suitable area (>0.9 probability) for *A. albopictus* and 92,779 km^2^ of highly suitable area for *D. v. virgifera*. The suitability prediction based on SM3 pseudo-absences identified no highly suitable locations for *A. albopictus* and a large 247,883 km^2^ area of highly suitable area for *D. v. virgifera*. Habitat suitability prediction based on the 3-step method (SM4) identified 8,752 km^2^ of highly suitable area for *A. albopictus* and 151,569 km^2^ for *D. v. virgifera.*

## Discussion

A number of studies have established that the pseudo-absence selection method used for SDMs affects model performance [2], [4], [10], [25], [72]. In this study, the effect of pseudo-absence selection methods on the performance of seven models was investigated. The results showed that methodological prescription of pseudo-absence points, similar to the 3-step method developed in this study, enhances model predictive power. The commonly used approaches are to constrain the background data geographically (similar to SM2), or environmental profiling of the background data (similar to SM3) [10], [20], [25]. However, some studies have reported that random pseudo-absence selection method (equivalent to SM1) works best in some contexts. For example, SM1 is considered to work well with logistic regression models [10] and when environmental data is too complex to perform environmental profiling [7]. Jiménez-Valverde *et al.* [2] and Lobo *et al.* [4] suggested that the best way to get potential distribution representation of a species is by using absences located relatively near the external boundary of the environmental domain and adding geographic proximity if the requirement is to get the realized distribution representation. In the three-step method, we quantified these boundaries by utilizing variable importance analysis over various distances from presence locations. The challenge was to maintain model performance while introducing spatial constraint on the potential background data. Environmentally profiled background data without any geographical constraint usually gives very high model AUC and sensitivity values because the data are overly and unrealistically discriminated. Rather than using an arbitrary distance, the 3-step pseudo-absence selection method utilizes an ecologically meaningful distance to specify geographic extent of background data, in order to minimize information loss due to the introduced spatial constraint. We found the optimum distance for the background data extent to be 350 km for the *A. albopictus* dataset and 3,000 km for *D. v. virgifera* dataset. Care should be taken not to associate distance obtained through variable importance analysis as a constant biogeographic characteristic of the species. The distance at which background data is bounded is identified based on the species relative area of occurrence. As a consequence, it is affected by the number of presence locations, their distribution and the extent of the study area. The identified distance must be re-calculated if the presence data or the extent of the study area changes.

### Variable Selection

Variable selection is an essential step in species distribution modelling. Selected variables and their relationship at the presence points are the mechanism by which ecological assumptions are incorporated in correlative species distribution models. Failing to select the appropriate explanatory variables leads to model results detached from ecological reality. In this study, we found large variation between the numbers and types of variables selected according to presence data and pseudo-absence selection method.

The between-species differences in the variables selected for each pseudo-absence scenario can be used to assess the effect of species presence data on variable selection. More variables were selected for the *A. albopictus* training dataset than *D. v. virgifera* in all pseudo-absence selection methods. This was because the *A. albopictus* dataset with 2,928 presence points covers a large area in geographic and environmental space, requiring more variables to characterise the training data than the *D. v. virgifera* dataset that has 64 presence points over a relatively limited geographic and environmental range. This result is not unexpected, the larger the environmental range of the species, the larger number of variables needed to construct a valid model.

The within-species differences in the variables selected show that pseudo-absence data has considerable influence on variable selection. A large number of variables in this case correspond to inconsistent pseudo-absence points that require a large number of variables to characterise the training data. The least number of variables were selected from data using the 3-step method (Table 1). More conservative variable selection is a result of a unique interplay of limiting background extent and robust environmental profiling used in the 3-step method, which excluded environmentally extreme outliers in the training data while providing clear environmental classification between presence and pseudo-absence points.

It is well established that the number of presences and the environmental data are critical for variable selection and accuracy of SDM predictions. However, defining appropriate unsuitable areas by selecting optimal pseudo-absences to contrast with suitable areas inferred from presence points is equally important.

### Model Performance

With respect to model kappa values, SM1 results show that random pseudo-absence selection method is not consistent either for the two species or the seven models tested. For example, the logistic regression model (LOG) performed well for *A. albopictus* with a high Kappa value but performed poorly for *D. v. virgifera*. This inconsistency is confirmed by Lobo *et al*. [4] who states that random pseudo-absence selection methods are unreliable due to their high dependence on species presence point distribution and abundance. High model performance using this method can occur by chance and is unlikely to be repeatable for different species or model scenarios as shown in this study. SM2 results were low for all models. Both SM1 and SM2 resulted in significantly low mean AUC and specificity scores compared with models using SM3 and SM4 pseudo-absences. SM1 and SM2, therefore, seem not ideal pseudo-absence selection methods to use in SDMs.

SM3 gave consistently high model performance (Kappa statistics) except for CTREE and CART models which had variable performance across the two species. The machine learning models using SM3 pseudo-absences performed consistently over the two species dataset. SM3 was found to perform well, especially for the LOG model giving similar high kappa values for both species. This result is despite reports stating that regression models work best under random selection methods [7], [10]. We attribute the good results from the LOG model on SM3 pseudo-absences to the use of a robust model (OCSVM) for environmental profiling of background data.

SM4 provided excellent kappa values for all models for the *D. v. virgifera* data set and 5 models of *A. albopictus* dataset. A single low kappa value was reported for the LOG model performance. There was no significant difference between AUC, sensitivity and specificity values between SM3 and SM4 methods despite that the background data for the pseudo-absence points of SM4 were geographically restricted. While there was no statistical difference, SM4 method achieves high model performance while avoiding extreme spatial and environmental locations that could lead to inconsistency in prediction for new areas.

### Model Consensus and Habitat Suitability

The highest percentage of predicted presences was obtained from the 3-step pseudo-absence selection method. This result is very important especially for invasive species studies where identifying potential areas suitable for the establishment for the target species is critical. The lowest predicted presence percentage was from the random selection method (SM1) both at a global and New Zealand scale. Comparisons of predicted presence maps were done to check consensus among models that used the same pseudo-absence method. We recognize that model consensus alone does not ensure high prediction accuracy because models can wrongly agree on the occurrence of a species. A good example is the high consensus among models using SM2 pseudo-absence points for prediction of *D. v. virgifera* distribution in New Zealand (Figure 6b), even when the Kappa model performance scores for these models were very low (Figure 4). However, high model consensus combined with high model performance scores is preferable to multiple models with high performance scores and low agreement. Furthermore, inconsistency between predictions makes SDM result interpretations difficult for decision makers. In this study, the three step method (SM4) provided the needed combination of high model performance in terms of Kappa values (Figure 4) and consistency in model predictions in terms of high model consensus (Figure 6a, b).

Habitat suitability predictions based on the 4 pseudo-absence types (Figure 2) gave different results in terms of the size and location of suitable areas for *A. albopictus* and *D. v. virgifera* (Figures 7, 8, 9, 10). Pseudo-absence points from SM1 and SM2 methods are not distinctly separated from presences in the environmental feature space (Figure 2a, b). This lack of discrimination is reflected in their respective habitat suitability predictions. Both SM1 and SM2 maps showed underestimation of the potential suitable area *for A. albopictus* and *D. v. virgifera* when overlaid with occurrence points. Pseudo-absences from both SM3 and SM4 methods were distinctly clustered away from presence points in the feature space allowing environmental discrimination (Figure 2c, d). Accordingly, most of the occurrence areas are identified by the SM3 and SM4 models as highly suitable for both species. While such high model sensitivity is beneficial to more accurately estimate the potential distribution of a species, it is possible to overestimate the potential distribution if highly discriminated presence/pseudo-absence training data are used [4]. Therefore, even if both SM3 and SM4 gave comparable suitability predictions, it is advisable to determine optimum background extent for pseudo-absence selection if the study area is at a global or regional scale.

### Implications for Future *A. albopictus* and *D. v. virgifera* Management in New Zealand

#### Aedes albopictus

The global distribution estimated for *A. albopictus* from SM1 and SM2 appropriately covered the native Southeast Asian and the introduced South American range, but did not cover the North American distribution accurately. The European and African population were also not accurately represented on the maps (Figure 7a, b). SM3 and SM4 global distribution maps for *A. albopictus* reflect the current complete range of *A. albopictus*. However, the extent of predicted suitable areas for *A. albopictus* in New Zealand varies between projections using SM3 and SM4 pseudo-absence methods. The SM3 projection (Figure 9c) only shows 2,000 km^2^ of moderately suitable area within New Zealand, whereas the SM4 projection identified over 8,000 km^2^ of highly suitable areas (Figure 9d). Given that other species from the *Aedes* genus have established in New Zealand and that *A. albopictus* is repeatedly intercepted at the New Zealand border [73], we suggest that the suitable areas identified by SM4 be considered in future mosquito related biosecurity assessments. The suitability projection difference between the SM3 and SM4 shows that incorporating a spatial dimension while environmental profiling has a significant effect on model predictions. *A. albopictus* is a particularly difficult species to model as it is currently undergoing a rapid range expansion. Previous studies showed that there is a niche shift throughout the dispersal history of *A. albopictus* [74]. It is important to select accurate presence and pseudo-absences data while projecting suitable areas for such species whose distribution spans a wide environmental range.

#### Diabrotica v. virgifera

Similar to *A. albopictus,* the SM1 and SM2 global species distribution model for *D. v. virgifera* did not fully reflect the current known distribution of the species (Figure 8a, b). The SM3 and SM4 predictions (Figure 8c, d) reflected the current known distribution, although the former was more conservative and the latter failed to characterize Central America, the native habitat of the species as highly suitable. An interesting variation in prediction of SM4 is the highly suitable areas identified close to East Africa, an area into which *D. v. virgifera* is expected to spread unless appropriate prevention measures are taken [75]. The SM4 suitability projection for *D. v. virgifera* in New Zealand showed northern and central areas of the North Island and areas east of the Southern Alps as highly suitable (Figure 10d). Although maize (*Zea mays*) production is not a major economic crop in New Zealand, it still accounts for 30% of the arable industry [76]. Biosecurity measures at the border are essential to prevent the entry of *D. v. virgifera,* a major maize pest, to New Zealand.

### Does Model Type Matter?

Several studies show that model type is a major source of uncertainty in SDM results [77], [78] among other factors like variable selection, data collinearity and pseudo-absence selection. Uncertainty in SDMs can also arise both from data inaccuracy and internal model error [79]. While little can be done by users to fix errors inherent in model algorithms, model error from data inaccuracy can be reduced by boosting input data quality. Models perform differently given different datasets (environmental data, presence data and pseudo-absence data). While the effect of the accuracy of environmental and presence data have been investigated in depth, the effect of accuracy of pseudo-absence points on model performance has been less investigated. In this study, we established that a robust pseudo-absence selection method can create an input dataset that improves the performance of the SDMs investigated here. That is shown by the low standard deviation in model results that used the 3-step (SM4) pseudo-absence points and the very high Kappa values. Well-structured training data with appropriate variables increases the performance of all models. However, it is still very important to choose models carefully while keeping presence data quality, environmental data and model expertise in mind.

### Advantages of the 3-step Pseudo-absence Selection Method

The advantages of the three-step pseudo-absence selection method proposed in this study are threefold. First, the variable importance analysis and background data limiting step (step 1) provide a balance between spatial and environmental information currently missing in pseudo-absence selection methods. Second, the use of the OCSVM for environmental profiling, instead of the current approaches that are unable to handle large variable datasets and complex non-linear relationships, provides an improved method to identify pseudo-absences in a complex environment. The proposed ensemble OCSVM framework is also important to avoid model over-fitting caused by highly discriminated training data. Third, the proposed use of k-means clustering to choose pseudo-absences instead of random selection from environmentally profiled data ensures not only environmentally dissimilar points are chosen but also provides a systematic way of obtaining a representative sample of the unsuitable environment. The other important advantage of the k-means clustering, compared with random sampling of environmentally profiled background data, is that results are more repeatable. This is essential, especially when performing ensemble modelling and climate change studies where standardised methods are required for appropriate replication.

The results show that spatial and environmental background data profiling before selecting pseudo-absence points is essential to increase prediction accuracy. Profiling is important because geo-environmentally profiled pseudo-absences have a clearer data structure and consistency than random, environmentally or spatially profiled pseudo-absence points. Clear data structure within pseudo-absence points means more information and less uncertainty during model training. More important, such detailed profiling of input data that simultaneously investigates geographical settings as well as environmental requirements should lead to greater understanding and the generation of interesting hypotheses about the relationship between species and their habitat that can be tested in future research.

### Caveats

The first step of the 3-step pseudo-absence selection system that identifies the appropriate distance within which background data is to be extracted can be quite time consuming and tedious. This can be overcome by developing an automated framework to test variable importance at a set of pre-set intervals.

Another concern is that a large number of presence points are available, coinciding with a small background extent in step 1. A small background extent that encompasses a large number of presence points may reduce the area available for environmental profiling at step 2. That could lead to a poorly discriminated environmental classification. That is not expected to be a common problem as accurate presence points are not usually available in abundance at a global or regional level. This, however, could be remedied by introducing a threshold that relates density of presence points to a minimum distance at which spatial extent of the background data is drawn.

### Conclusion

When the complete range of a species is unknown, visualizing the distribution of the known presence locations both in geographic and environmental space and assessing the species ROA, is valuable. If presence data is highly clustered both in geographical and environmental space, using presence-only models often leads to extrapolation. In such cases, it is advisable to use presence-absence models with a pseudo-absence selection method that considers both the spatial and environmental space [2], [4]. When performing species distribution modelling for species undergoing rapid range expansion with dynamic presence data records, new distances should be re-calculated to specify background data geographic extent with the addition of new presence points according to variable importance analysis over various distances from the new presence dataset.

The three-step pseudo-absence selection method (SM4) was shown to result in high model performance while spatially constraining background data to filter out extreme geographically dissimilar locations. Any loss of information from bounding background data geographically before environmental profiling is compensated by the added precision resulting from reduced over-fitting of an SDM model. While this result holds for the models tested in this study, further investigation over more species and models is recommended.

## Supporting Information

Figure S1 **Boundaries of background datasets extracted from circular buffers drawn at various radii from** ***D. v. virgifera*** **presence points.** The bold red boundary shows the optimum background extent identified by the variable importance analysis.
(TIF)

## References

[1] Araújo MB, Peterson AT (2012) Uses and misuses of bioclimatic envelope modelling. Ecology 93: 1527–1539. 22919900 10.1890/11-1930.1

[2] Jiménez-Valverde A, Lobo JM, Hortal J (2008) Not as good as they seem: the importance of concepts in species distribution modelling. Diversity and Distributions 14: 885–890.

[3] Phillips SJ, Anderson RP, Schapire RE (2006) Maximum entropy modeling of species geographic distributions. Ecological Modelling 190: 231–259.

[4] Lobo JM, Jiménez-Valverde A, Hortal J (2010) The uncertain nature of absences and their importance in species distribution modelling. Ecography 33: 103–114.

[5] Peterson AT (2006) Uses and Requirements of Ecological Niche Models and Related Distributional Models. Biodiversity Informatics 3: 59–72.

[6] Kearney M, Porter W (2009) Mechanistic niche modelling: combining physiological and spatial data to predict species’ ranges. Ecology Letters 12: 334. 19292794 10.1111/j.1461-0248.2008.01277.x

[7] Wisz M, Guisan A (2009) Do pseudo-absence selection strategies influence species distribution models and their predictions? An information-theoretic approach based on simulated data. BMC Ecology 9: 8. 19393082 10.1186/1472-6785-9-8 PMC2680809

[8] Soberon J, Peterson AT (2005) Interpretation of models of fundamental ecological niches and species’ distriputional areas. Biodiversity Informatics 2: 1–10.

[9] Hirzel AH, Hausser J, Chessel D, Perrin N (2002) Ecological-Niche factor analysis: How to compute habitat-suitability maps without absence data? Ecology 83: 2027.

[10] Barbet-Massin M, Jiguet F, Albert CH, Thuiller W (2012) Selecting pseudo-absences for species distribution models: how, where and how many? Methods in Ecology and Evolution 3: 327–338.

[11] Busby JR (1986) A biogeoclimatic analysis of Nothofagus cunninghamii (Hook.) Oerst. in southeastern Australia. Australian Journal of Ecology 11: 1–7.

[12] Carpenter G, Gillison AN, inter JW (1993) DOMAIN: a flexible modelling procedure for mapping potential distributions of plants and animals. Biodiversity and Conservation 2: 667–680.

[13] Li W, Guo Q, Elkan C (2011) Can we model the probability of presence of species without absence data? Ecography 34: 1096–1105.

[14] Phillips SJ, Dudík M (2008) Modeling of species distributions with Maxent: new extensions and a comprehensive evaluation. Ecography 31: 161–175.

[15] Elith J, Graham CH, Anderson RP, Dudik M, Ferrier S, et al (2006) Novel methods improve prediction of species; distributions from occurance data. Ecography 29: 129–151.

[16] Lorena AC, Jacintho LFO, Siqueira MF, Giovanni RD, Lohmann LG, et al (2011) Comparing machine learning classifiers in potential distribution modelling. Expert Systems with Applications 38: 5268–5275.

[17] Poulos HM, Chernoff B, Fuller PL, Butman D (2012) Ensemble forecasting of potential habitat for three invasive fishes. Aquatic Invasions 7: 59–72.

[18] Elith J, Leathwick J (2007) Predicting species distributions from museum and herbarium records using multiresponse models fitted with multivariate adaptive regression splines. Diversity and Distributions 13: 265–275.

[19] Hirzel, A H, Heifer V, Metral F (2001) Assessing habitat-suitability models with a virtual species. Ecological Modelling 145: 111–121.

[20] Zaniewski AE, Lehmann A, Overton JM (2002) Predicting species spatial distributions using presence-only data: a case study of native New Zealand ferns. Ecological Modelling 157: 261–280.

[21] Hastie T, Fithian W (2013) Inference from presence-only data; the ongoing controversy. Ecography: 864–867. 10.1111/j.1600-0587.2013.00321.x PMC4258395 25492992

[22] Brotons L, Thuiller W, Araújo MB, Hirzel AH (2004) Presence-absence versus presence-only modelling methods for predicting bird habitat suitability. Ecography 27: 437–448.

[23] Manevitz LM, Yousef M (2002) One-class svms for document classification. J Mach Learn Res 2: 139–154.

[24] VanDerWal J, Shoo Luke P, Graham C, Williams Stephen E (2009) Selecting pseudo-absence data for presence-only distribution modeling: How far should you stray from what you know? Ecological Modelling 220: 589–594.

[25] Chefaoui RM, Lobo JM (2008) Assessing the effects of Pseudo-absence on predictive distribution model performance. Ecological Modelling 210: 478–486.

[26] Warton DI, Shepherd LC (2010) Poisson point process models solve the “pseudo-absence problem” for presence-only data in ecology. Ann Appl Stat 4: 1383–1402.

[27] LÜTolf M, Kienast F, Guisan A (2006) The ghost of past species occurrence: improving species distribution models for presence-only data. Journal of Applied Ecology 43: 802–815.

[28] Stockwell D, Peters D (1999) The GARP modelling system: Problems and solutions to automated spatial prediction. International Journal of Geographical Information Science 13: 143–158.

[29] Engler R, Guisan A, Rechsteiner L (2004) An improved approach for predicting the distribution of rare and endangered species from occurrence and pseudo-absence data. Journal of Applied Ecology 41: 263–274.

[30] Lobo JM, Verdú JR, Numa C (2006) Environmental and geographical factors affecting the Iberian distribution of flightless Jekelius species (Coleoptera: Geotrupidae). Diversity and Distributions 12: 179–188.

[31] Farber O, Kadmon R (2003) Assessment of alternative approaches for bioclimatic modeling with special emphasis on the Mahalanobis distance. Ecological Modelling 160: 115–130.

[32] Hengl T (2009) A Practical Guide to Geostatistical Mapping. Open Access Publication. Available: http://spatial-analyst.net/book/system/files/Hengl_2009_GEOSTATe2c1w.pdf Accessed 2012 April 15: 291.

[33] Roiz D, Neteler M, Castellani C, Arnoldi D, Rizzoli A (2011) Climatic Factors Driving Invasion of the Tiger Mosquito (*Aedes albopictus*) into New Areas of Trentino, Northern Italy. PLoS ONE 6: e14800. 21525991 10.1371/journal.pone.0014800 PMC3078124

[34] Gratz NG (2004) Critical review of the vector status of Aedes albopictus. Medical and Veterinary Entomology 18: 215–227. 15347388 10.1111/j.0269-283X.2004.00513.x

[35] Honório NA, Silva WdC, Leite PJ, Gonçalves JM, Lounibos LP, et al (2003) Dispersal of Aedes aegypti and Aedes albopictus (Diptera: Culicidae) in an urban endemic dengue area in the State of Rio de Janeiro, Brazil. Memórias do Instituto Oswaldo Cruz 98: 191–198. 10.1590/s0074-02762003000200005 12764433

[36] Coats SA, Tollefson JJ, Mutchmor JA (1986) Study of migratory flight in the Western Corn Rootworm (Coleoptera: Chrysomelidae). Environmental Entomology 15.

[37] Ciosi M, Miller NJ, Kim KS, Giordano R, Estoups A, et al (2008) Invasion of Europe by the western corn rootworm, *Diabrotica virgifera virgifera*: multiple transatlantic introductions with various reductions of genetic diversity. Molecular Ecology 17: 3622–3625. 10.1111/j.1365-294X.2008.03866.x 18662220

[38] Henmerik L, Busstra C, Mols P (2004) Predicting the temprature-dependent natural population expansion of the western corn rootworm, Diabrotica virgifera. Entomologia Expermimentalis et Applicata 111: 59–69.

[39] Miller N, Estoup A, Toepfer S, Bourguet D, Lapchin L, et al (2005) Muliple transatlantic introductions of the western corn rootworm. Science 310: 992. 16284172 10.1126/science.1115871

[40] Moeser J, Vidal S (2004) Do alternative host plants enhance the invasion of maize pest Diabrotica virgifera virgifera (coleoptera: Chrysomelidae, Galerucinae) in Europe. Environmental Entomology 33: 1170, 1174–1176.

[41] Onstad DW, Crwoder DW, Isard SA, Levine E, Spencer JL, et al (2003) Does landscape diversity slow the spread of rotation-resistant Western Corn Rootworm (Coleoptera: Chyrysomelidae)? Environmental Entomology 32: 992–1001.

[42] Toepfer S, Levay N, Kiss J (2006) Adult movements of newly introduced alien *Diabrotica virgifera virgifera* (Coleoptera: Chrysomelidae) from non-host habitats. Bulletin of Entomological Research 96: 327–335. 16923199

[43] Hijmans RJ, Cameron S, Parra J (2005) BIOCLIM. Available: http://www.worldclim.org/bioclim Accessed 2011 May 4.

[44] Hijmans RJ, Cameron SE, Parra JL, Jones PG, Jarvis A (2005) Very high resolution interpolated climate surfaces for global land areas. International Journal of Climatology 25: 1965–1978.

[45] NASA-GSFC (2000) EOS data products handbook. In: Parkinson CL, Greenstone R, editors. Greenbelt, Maryland: National Aeronautics and Space Administration (NASA) Goddard Space Flight Center. Available: http://eospso.gsfc.nasa.gov/ftp_docs/data_products_vol2.pdf Accessed 2010 Dec 22.

[46] EROS (1996) GTOPO30. In: Center ED, editor. Sioux Falls. Available: http://eros.usgs.gov/#/Find_Data/Products_and_Data_Available/gtopo30_info Accessed 2012 Jan 22.

[47] Schölkopf B, Platt JC, Shawe-Taylor J, Smola AJ, Williamson RC (2001) Estimating the support of a high-dimensional distribution. Neural computation 13: 1443–1471. 11440593 10.1162/089976601750264965

[48] Tuv E, Borisov A, Runger G, Torkkola K (2009) Feature selection with ensembles, artificial variables, and redundancy elimination. The Journal of Machine Learning Research 10: 1341–1366.

[49] Kleinbaum DG, Klein M (2005) Logistic regression. New York: Springer.

[50] Venables W, Ripley BD (1997) Modern applied statistics with S-plus. New York: Springer-Verlag. 548 p.

[51] Hothorn T, Hornik K, Zeileis A (2006) Unbiased recursive partitioning: A conditional inference framework. J of Comput Graph Stat 15: 651–674.

[52] Ripley BD (1994) Neural Networks and Related Methods for Classification. Journal of the Royal Statistical Society Series B (Methodological) 56: 409–456.

[53] McCallum A, Nigam K. A comparison of event models for naive bayes text classification; 1998. AAAI Press. 41–48.

[54] Vapnik VN (1995) The nature of statistical learning theory. New York: Springer-Verlag.

[55] Haykin S (1998) Neural networks: A comprehensive foundation Prentice Hall.

[56] Breiman L (2001) Random Forests. Machine Learning 45: 5–32.

[57] Jiménez-Valverde A, Lobo JM (2007) Threshold criteria for conversion of probability of species presence to either–or presence–absence. Acta Oecologica 31: 361–369.

[58] Manel S, Williams HC, Ormerod SJ (2001) Evaluating presence–absence models in ecology: the need to account for prevalence. Journal of Applied Ecology 38: 921–931.

[59] R Core Team (2012) R: A language and environment for statistical computing. Vienna, Austria: R Foundation for Statistical Computing.Available: http://www.R-project.org/Accessed 2012 Oct 29.

[60] Mendiburu Fd (2012) agricolae: statistical procedures for agricultural research R package version 1.1–2. Available: http://CRAN.R-project.org/package=agricolae Accessed 2012 Sep 12.

[61] Venables WN, Ripley BD (2002) Modern applied statistics with S.. New York: springer.

[62] Hothorn T, Hornik K, Wiel MAvd, Zeilleis A (2006) A lego system for condditional inference. The American Statistician 60: 257–263.

[63] Meyer D, Dimitriadou E, honik K, Leisch F, Weingessel A (2007) e1071: Misc functions of the department of statistics (e1071). R package 1.5–17 ed. Available: http://cran.r-project.org/web/packages/e1071/index.html Accessed 2012 Jun 13.

[64] Karatzoglou A, Smola A, Hornik K, Zeileis A (2004) Kernlab-An S4 package for kernel methods in R. Journal of statistical software. 11: 1–20.

[65] Weihs C, Ligges U, Luebke K, Raabe N (2005) klaR Analyzing german business cycles. In: Baier D, Decker R, Schmidt-thieme L, editors. Data analysis and decision support. Berlin: Springer-Verlag. 335–343.

[66] Hothorn T, Bretz F, Westfall P (2008) Simultaneous inference in general parametric models. biometrical journal 50: 346–363. 18481363 10.1002/bimj.200810425

[67] Liaw A, Wiener M (2002) Classification and Regression by randomForest. Available: http://cran.r-project.org/web/packages/randomForest/index.html Accessed 2012 Jun 13.

[68] Pebesma EJ, Bivand RS (2005) SP: Classses and methods for spatial data in R. Available: http://cran.r-project.org/web/packages/sp/index.html Accessed 2012 Jun 13.

[69] Diaz-Uriarte R (2009) varSelRF: Variable selection using random forests. R package version 0.7–1. ed. Available: http://ligarto.org/rdiaz/Software/Software.html Accessed 2012 Jun 13.

[70] MathWorks (2011) MATLAB. 7.12.0.635 ed. Massachusetts: The MathWorks Inc. Available: http://www.mathworks.com.au Accessed 2012 Jun 13.

[71] ESRI (2010) ArcMap. 10.0 ed. Redlands, CA: Environmental Systems Research Institute. Available: http://www.esri.com/Accessed 2011 May 02.

[72] Thuiller W, Brotons L, Araújo MB, Lavorel S (2004) Effects of restricting environmental range of data to project current and future species distributions. Ecography 27: 165–172.

[73] Derraik JGB (2004) Exotic mosquitoes in New Zealand: a review of species intercepted, their pathways and ports of entry. Australian and New Zealand Journal of Public Health 28: 433–444. 15707185 10.1111/j.1467-842x.2004.tb00025.x

[74] Medley KA (2009) Niche shifts during the global invasion of the Asian tiger mosquito, Aedes albopictus Skuse (Culicidae), revealed by reciprocal distribution models. Global Ecology and Biogeography 19: 122–133.

[75] Hummel HE, Dinnesen S, Nedelev T, Modic S, Urek G, et al (2008) *Dibrotica virgifera virgifera* LeConte in confrontation mood: simultaneous geographical and host spectrum expansion in southeastern Slovenia. MittDtsch GesAllg Ent 16: 127–128.

[76] Barber A, Pellow G, Barber M (2011) Carbon Footprint of New Zealand Arable Production – Wheat, Maize Silage, Maize Grain and Ryegrass Seed. Ministry of Agriculture and Forestry.Available: http://www.fedfarm.org.nz/Files/2011-MPIGrainCarbon.pdf Accessed 30 Oct 2012.

[77] Buisson L, Thuiller W, Casajus N, Lek S, Grenouillet G (2010) Uncertainty in ensemble forecasting of species distribution. Global Change Biology 16: 1145–1157.

[78] Dormann CF, Purschke O, Marquez JRG, Lautenbach S, Schroder B (2008) Components of uncertainity in species distribution analysis: A case study of the Great Grey Shrike. Ecology 89: 3371–3386. 19137944 10.1890/07-1772.1

[79] Elith J, Leathwick JR (2009) Species Distribution Models: Ecological Explanation and Prediction Across Space and Time. Annual Review of Ecology, Evolution, and Systematics 40: 677–697.

