# Supplementary figures and images for: Novel Three-Step Pseudo-Absence Selection Technique for Improved Species Distribution Modelling
Source: PLoS One. 2013 Aug 13;8(8):e71218. doi: 10.1371/journal.pone.0071218 (PMC3742778; doi:10.1371/journal.pone.0071218)

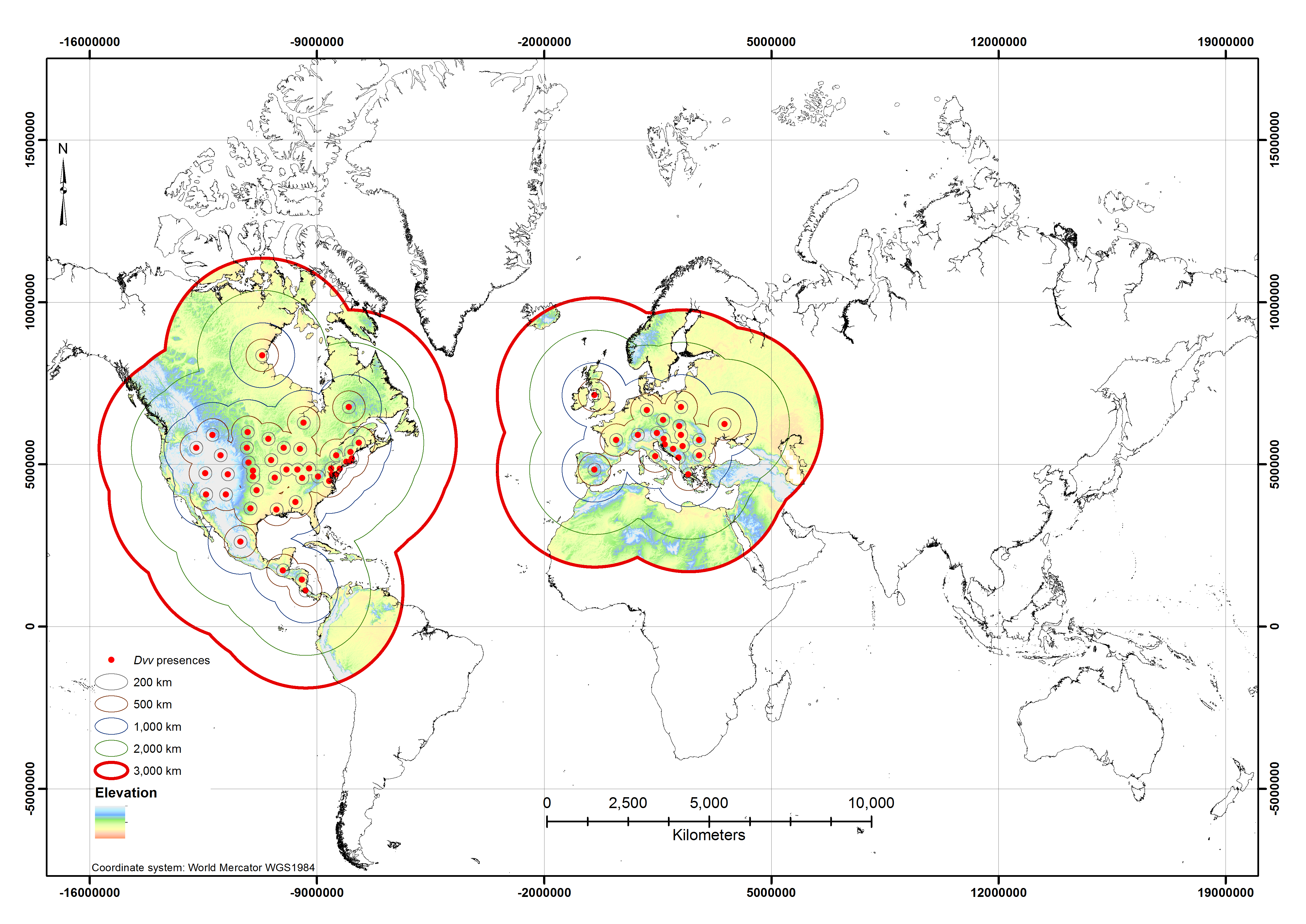

Supplement: Figure S1 — Boundaries of background datasets extracted from circular buffers drawn at various radii from D. v. virgifera presence points. The bold red boundary shows the optimum background extent identified by the variable importance analysis. (TIF) [file pone.0071218.s001.tif]
